# Yoga for Chemotherapy-Induced Peripheral Neuropathy and Fall Risk: A Randomized Controlled Trial

**DOI:** 10.1093/jncics/pkaa048

**Published:** 2020-06-04

**Authors:** Ting Bao, Iris Zhi, Raymond Baser, Madeline Hooper, Connie Chen, Lauren Piulson, Qing S Li, Mary Lou Galantino, Victoria Blinder, Mark Robson, Andrew Seidman, Katherine S Panageas, Jun J Mao

**Affiliations:** Memorial Sloan Kettering Cancer Center, New York, NY, USA; Memorial Sloan Kettering Cancer Center, New York, NY, USA; Memorial Sloan Kettering Cancer Center, New York, NY, USA; Memorial Sloan Kettering Cancer Center, New York, NY, USA; Memorial Sloan Kettering Cancer Center, New York, NY, USA; Memorial Sloan Kettering Cancer Center, New York, NY, USA; Memorial Sloan Kettering Cancer Center, New York, NY, USA; Stockton University, Galloway, NJ, USA; Memorial Sloan Kettering Cancer Center, New York, NY, USA; Memorial Sloan Kettering Cancer Center, New York, NY, USA; Memorial Sloan Kettering Cancer Center, New York, NY, USA; Memorial Sloan Kettering Cancer Center, New York, NY, USA; Memorial Sloan Kettering Cancer Center, New York, NY, USA

## Abstract

**Background:**

Chemotherapy-induced peripheral neuropathy (CIPN) is a common, debilitating side effect that worsens quality of life and increases the risk of falls in cancer survivors. Evidence of yoga’s safety and efficacy in treating CIPN is lacking.

**Methods:**

In a randomized controlled study, we assigned breast and gynecological cancer survivors with persistent moderate-to-severe CIPN pain, numbness, or tingling with a score of 4 or greater (0-10 numeric rating scale [NRS]) for at least 3 months after chemotherapy to 8 weeks of usual care or yoga focused on breathwork and musculoskeletal conditioning. Primary endpoint was treatment arm differences for NRS, and secondary endpoints were Functional Assessment of Cancer Therapy/Gynecologic Oncology Group-Neurotoxicity subscale (FACT/GOG-Ntx), and Functional Reach Test after week 8. We tested treatment arm differences for each outcome measure using linear mixed models with treatment-by-time interactions. All statistical tests were two-sided.

**Results:**

We randomly assigned 41 participants into yoga (n = 21) or usual care (n = 20). At week 8, mean NRS pain decreased by 1.95 points (95% confidence interval [CI] = -3.20 to -0.70) in yoga vs 0.65 (95% CI = -1.81 to 0.51) in usual care (*P* = .14). FACT/GOG-Ntx improved by 4.25 (95% CI = 2.29 to 6.20) in yoga vs 1.36 (95% CI = -0.47 to 3.19) in usual care (*P* = .035). Functional reach, an objective functional measure predicting the risk of falls, improved by 7.14 cm (95% CI = 3.68 to 10.59) in yoga and decreased by 1.65 cm (95% CI = -5.00 to 1.72) in usual care (*P* = .001). Four grade 1 adverse events were observed in the yoga arm.

**Conclusion:**

Among breast and gynecological cancer survivors with moderate-to-severe CIPN, yoga was safe and showed promising efficacy in improving CIPN symptoms.

Chemotherapy-induced peripheral neuropathy (CIPN) is a common and dose-limiting side effect of neurotoxic chemotherapy (ie, taxanes, vinca alkaloids, platinum, and bortezomib) that interferes with patients’ daily function and worsens quality of life (1–3). In a study of 512 cancer survivors, 47% reported persistent neuropathy up to 6 years after chemotherapy completion. Furthermore, these survivors exhibited altered gait patterns with slower and shorter steps, as well as a fall risk 1.8-fold greater than that of those without CIPN (4). In another study, 12% of cancer survivors with CIPN reported falls within a 3-month period (5). These evidences highlight the need for an effective treatment for CIPN to improve quality of life and safety among cancer survivors. Current empirical treatments for CIPN include symptom management with analgesics, antidepressants, and antiepileptics (6). However, these approaches are limited not only by toxicities but also by patients’ reluctance to further medicate a medication-related condition.

Yoga is a meditative movement therapy that improves body conditioning, flexibility, and balance through mind-body awareness. A myriad of pilot and feasibility studies suggest that yoga may help improve quality of life (7–13), anxiety (9,14–16), depression (8,9,14–18), fatigue (13,17–20), and functional outcomes (13,21,22) in breast cancer patients and survivors who received chemotherapy (23). Although CIPN can substantially impact function and increase the risk of falls among cancer patients, only 2 small single arm (N = 10) and randomized controlled studies (N = 26, randomized to yoga, Reiki, meditation, or educational control) have evaluated the effects of yoga on improving CIPN symptoms and reducing CIPN-related falls in cancer patients (24,25).

We conducted a 2-arm randomized wait-list controlled trial in breast and gynecological cancer survivors with moderate-to-severe CIPN to assess the preliminary safety and effectiveness of using yoga to improve CIPN symptoms and functional outcomes that are predictive of fall risks.

## Methods

### Study Participants

Following approval by the Memorial Sloan Kettering Cancer Center (MSK) Institutional Review Board (ClinicalTrials.gov Identifier: NCT03292328), we recruited participants between February 2018 and May 2019 at MSK in Manhattan. Eligibility requirements included English-speaking cancer survivors age 18 or older with a primary diagnosis of stage I-III breast, ovarian, uterine, or endometrial cancer who completed neurotoxic chemotherapy (eg, paclitaxel, docetaxel, carboplatin) at least 3 months before enrollment; reported moderate-to-severe CIPN, defined as tingling, numbness, or pain rated 4 or greater on the 11-point numerical rating scale (NRS); and maintained an Eastern Cooperative Oncology Group (ECOG) performance status of 0-2. We excluded patients with metastatic disease and those practicing yoga or receiving physical therapy. We included patients on antineuropathy medication if the regimen had been stable for the past 3 months and could be maintained throughout the study. Informed consent was obtained before participant registration. MSK’s clinical research database was used to randomly assign participants (1:1 ratio) into yoga and usual care arms stratified by severity of the baseline symptom (moderate, 4-6 vs severe, 7-10 on NRS).

### Study Design and Intervention

The yoga group practiced 60 minutes of yoga daily for 8 weeks, which is the suggested length of practice based on previous yoga studies on cancer patients (21,26,27). The 8-week yoga intervention included in-person group classes twice a week and at-home practice via a study-provided video on days that group classes were not held (5 times per week). The usual care group, the wait-list control arm, did not receive any interventions throughout the 12 weeks. In the case of treatment discontinuation, we made all attempts to have participants complete assessments up to week 12.

The yoga protocol for this trial emphasized breathwork (*pranayama*) to engage the parasympathetic nervous system and modifiable postures (*asanas*) to improve musculoskeletal flexibility, strength, and balance (21,26–30). Similar to other yoga- and exercise-based CIPN and fall interventions, the selected *asanas* focused on increasing parasympathetic activation, circulation and aerobic capacity, joint strength and mobility, spinal flexibility and tone, core strength, balance, and proprioception (21,25,26,31–37).

### Outcomes

We assessed yoga and usual care participants at baseline, and at weeks 4, 8, and 12. The primary endpoint was 3 aspects of patients’ CIPN symptomatology: pain, numbness, and tingling, as scored by the NRS at 8 weeks. Secondary endpoints include the Functional Assessment of Cancer Therapy/Gynecologic Oncology Group-Neurotoxicity subscale (FACT/GOG-Ntx), functional reach, and chair to stand. Participants were required to keep a home practice diary in which they recorded pain medication usage and changes, home yoga practice adherence, and intervention-related adverse events that they reviewed with a research assistant at each time point. Assessors were not blinded to allocation of participants. We applied the Common Terminology Criteria for Adverse Events (CTCAE v4.0) to document adverse events in home diaries.

CIPN symptom severity was rated on a reliable and validated 11-point NRS used for measuring intensity (0-10 scale with higher scores representing greater severity) (38–41). Each symptom (tingling, numbness, and pain) was rated separately.

We administered the 11-item Neurotoxicity subscale of the FACT/GOG-Ntx questionnaire to assess neuropathy-related quality of life. With demonstrated clinical validity and sensitivity to longitudinal symptom changes, this tool assesses sensory, motor, and auditory neuropathy, and dysfunction associated with neuropathy (42,43). Cumulative scores exist within a 0-44 range, with higher scores indicating better quality of life.

The Functional Reach Test assesses stability and balance by measuring the maximum distance an individual can reach forward while standing in a fixed position. Participants were instructed to flex the test arm to 90˚ and reach forward as far as possible before taking a step. The reach is determined by the total excursion of the third metacarpal from the starting point (with the hand held in a fist) to the point just before balance is lost. This was repeated 3 times with the average used as the final score (44,45). Functional reach has been shown to predict fall recurrence (46,47), with no reach ability posing an eightfold likelihood of falling compared with those who can reach over 25.4 cm (44,46). It has shown criterion validity, predictive validity, test-retest reliability, and interobserver reliability for younger and older adults (48), and possesses attributes for meaningful balance assessment (49), especially in the presence of CIPN. During assessment, research staff remained in close proximity to patients to ensure safety in the case of loss of balance.

Chair to stand is a standardized physical performance test (50,51). The ability to stand up without assistance is important for independent living and fall prevention (52,53). Participants were instructed to stand up from a chair and sit back down as quickly as they could 5 times. Total time used was recorded in seconds, with a longer time indicating worse performance. Chair to stand has been shown to predict recurrent risk of falls (relative risk = 1.74) (54,55).

Patients were instructed to walk at their usual pace from a standing position behind a starting line to a finish line 4 meters away. We recorded the time beginning at the first foot movement and ending when a foot completely crosses the finish line. Results are reported as gait speed in meters per second (56,57).

As prespecified in the study protocol, a sample size of 36 gives a 95% confidence interval (CI) around the estimate of the difference between means of +/- 0.58 standard deviations (SDs) of the postyoga CIPN symptom severity. Margining a 10% dropout rate, we recruited 20 subjects per arm (total of 40 subjects) to fall within the precision of our sample size calculation. To estimate potential treatment effects and provide insight into symptom and function trajectories over time, while also including patients with missing follow-up scores in the analysis per the intention-to-treat principle, we analyzed each outcome measure using linear mixed models (LMMs). These LMMs included data from all 4 assessment times (baseline, weeks 4, 8, and 12) and from all patients with a nonmissing baseline measure. We included assessment time, treatment arm, and the interaction between assessment time and treatment arm in the models. This allowed us to estimate mean changes for each arm relative to baseline, as well as test for differences between arms in change from baseline at each follow-up time. From these LMM models, for each outcome we calculated the model-based means and 95% confidence intervals by arm and assessment time and used a series of contrasts to test for statistically significant within-arm changes from baseline as well as between-arm differences in changes from baseline. Our prespecified primary endpoint was the difference between arms in NRS change from baseline to week 8; however, we present the results from all study assessment times for completeness. To ascertain study feasibility, we calculated the accrual rate with a 95% Poisson confidence interval and the proportion of patients who completed their 8-week questionnaires with a 95% confidence intervals.

## Results

### Patient Characteristics

**Figure 1.**
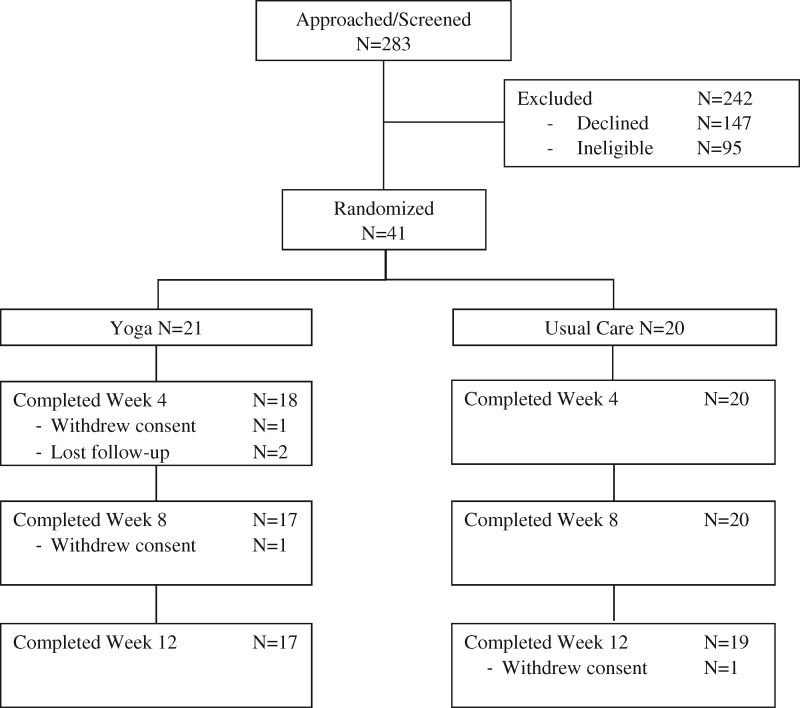
Study CONSORT diagram.

The study CONSORT diagram is depicted in Figure 1. Of the 283 cancer survivors screened, 147 declined and 95 were ineligible. A total of 41 patients were enrolled and randomized into yoga (n = 21) and usual care (n = 20) arms. Patient characteristics are listed in Table 1. Patients were balanced between 2 arms, although the yoga arm had more patients who received non-paclitaxel alone chemotherapy compared with the usual care arm 33.3% vs 5.0% (Table 1). There were no differences between the randomly allocated treatment groups in baseline scores for pain, numbness, or tingling.

**Table 1.** Patient characteristics^a^

Characteristics	Overall (N = 41)	Yoga (n = 21)	UC (n = 20)
Median patient age, y (min, max)	61.7 (35.5, 79.0)	60.0 (35.5, 77.9)	62.3 (42.4, 79.0)
Median body mass index, (min, max)	26.6 (17.8, 35.9)	26.6 (18.7, 35.5)	26.5 (17.8, 35.9)
Race, No. (%)			
White	23 (56.1	11 (52.4)	12 (60.0)
Black	8 (19.5)	4 (19.0)	4 (20.0)
Asian	5 (12.2)	4 (19.0)	1 (5.0)
Unknown	5 (12.2)	2 (9.5)	3 (15.0)
Ethnicity, No. (%)			
Hispanic	2 (4.9)	1 (4.8)	1 (5.0)
Non-Hispanic	39 (95.1)	20 (95.2)	19 (95.0)
Cancer type, No. (%)			
Breast	38 (92.7)	18 (85.7)	20 (100.0)
Uterine	2 (4.9)	2 (9.5)	0 (0.0)
Ovarian	1 (2.4)	1 (4.8)	0 (0.0)
Cancer stage, No. (%)			
Stage I	11 (26.8)	6 (28.6)	5 (25.0)
Stage II	15 (36.6)	5 (23.8)	10 (50.0)
Stage III	13 (31.7)	9 (42.9)	4 (20.0)
Other	2 (4.9)	1 (4.8)	1 (5.0)
Median years since diagnosis, (min, max)	3.9 (0.9, 25.8)	3.5 (0.9, 25.8)	4.1 (1.3, 15.8)
Median years since CTx ended, (min, max)	3.1 (0.5, 15.3)	3.1 (0.5, 10.4)	3.7 (0.9, 15.3)
Type of CTx, No. (%)			
Carboplatin	1 (2.4)	1 (4.8)	0 (0.0)
Docetaxel	2 (4.9)	2 (9.5)	0 (0.0)
Docetaxel & carboplatin	3 (7.3)	2 (9.5)	1 (5.0)
Paclitaxel	33 (80.5)	14 (66.7)	19 (95.0)
Paclitaxel & carboplatin	2 (4.9)	2 (9.5)	0 (0.0)
Median baseline NRS (min, max)			
Pain	−	4.10 (3.0, 5.2)	3.40 (2.3, 4.6)
Numbness	−	5.14 (4.0, 6.3)	5.05 (3.8, 6.3)
Tingling	−	4.33 (3.1, 5.6)	3.50 (2.2, 4.8)
Pain medication use	−	15 (71)	14 (70)

^a^CTx = chemotherapy; NRS = numeric rating scale; UC = usual care.

### Treatment Adherence

Enrollment was open for 15 months at an accrual rate of 2.7 (95% CI = 2.0 to 3.7) patients per month. In the yoga arm, 16 (95% CI = 76.2%, 54.9% to 89.4%) subjects completed the outcome assessments at week 8 and 17 (95% CI = 81.0%, 60.0% to 92.3%) at week 12. In the usual care arm, 20 (95% CI = 100.0%, 83.9% to 100.0%) completed the outcome assessments at week 8 and 19 (95% CI = 95.0%, 76.4% to 99.1%) at week 12. Overall, the percentage of patients completing the week 8 and 12 assessments was 87.8% (95% CI = 74.5% to 94.7%) for each assessment. Participants in the usual care group dropped out of the study because of loss of job and unwillingness to complete continuous assessments. Participants in the yoga group dropped out because of dissatisfaction with the intensity of the yoga regimen, long commute to the study site, and work-related conflict; 1 patient was lost to follow-up.

### CIPN Symptom Outcomes

**Figure 2.**
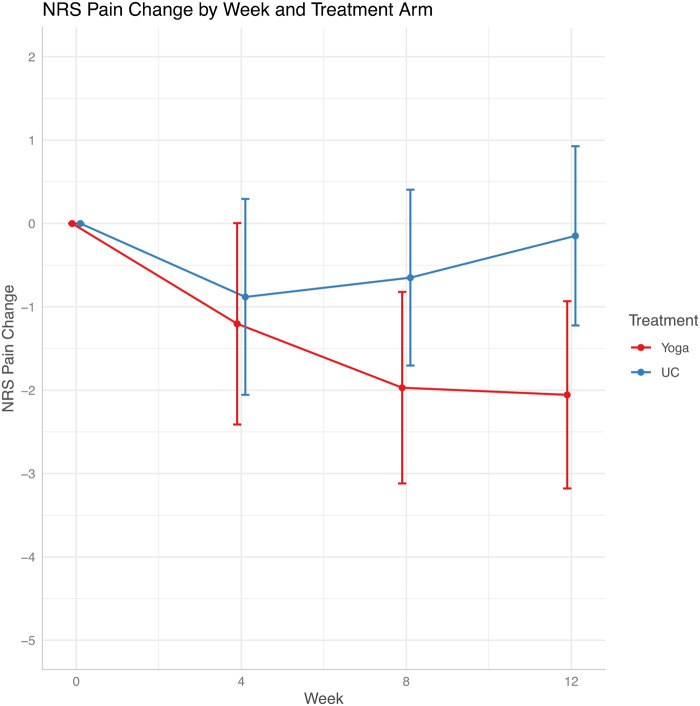
Changes in NRS score by weeks. NRS = numeric rating scale; UC = usual care.

**Figure 3.**
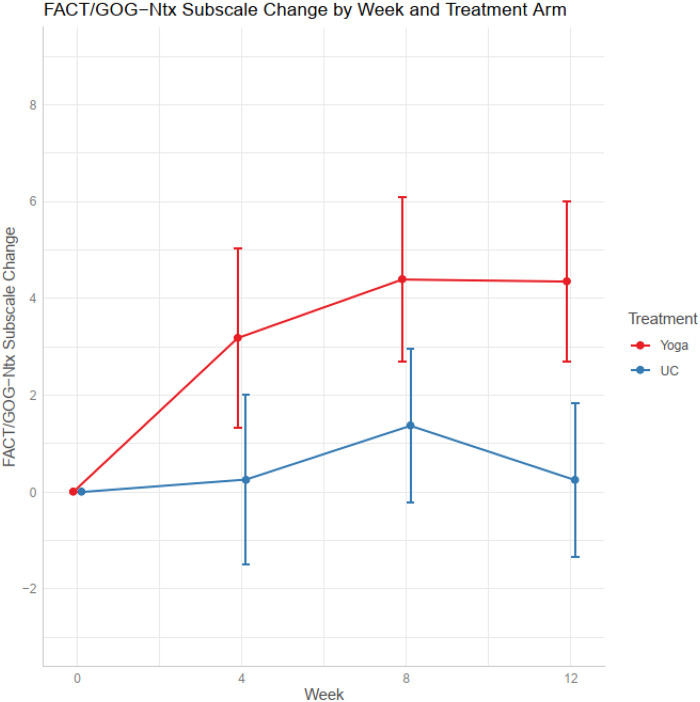
Changes in FACT/GOG-Ntx subscale score by weeks. FACT/GOG-Ntx = Functional Assessment of Cancer Therapy/Gynecologic Oncology Group-Neurotoxicity; UC = usual care.

At week 8 from baseline, mean NRS pain decreased by 1.95 points (95% CI = -3.20 to -0.70) in the yoga arm, and by 0.65 points (-1.81, 0.51) in the usual care arm; *P* = .14. At week 12 from baseline, mean NRS pain decreased by 2.03 points (95% CI = -3.25 to -0.80) in the yoga arm, and by 0.08 points (95% CI = -1.27 to 1.10) in the usual care arm; *P* = .026 (Figure 2 and Table 2). No statistically significant change was observed in NRS numbness, tingling, or total NRS score for yoga vs usual care arms (Table 2). At week 8, the FACT/GOG-Ntx subscale increased by 4.25 (95% CI = 2.29 to 6.20) in the yoga group vs 1.36 (95% CI = -0.47 to 3.19) in the usual care group; *P* = .035 (Figure 3 and Table 2).

**Table 2.** Within- and between-arm comparisons of outcomes over time^a^

Outcome	Week	Yoga arm			UC arm			Differences in change from baseline (yoga-UC), Mean (95% CI)	*P* value of the difference
		n	Mean (95% CI)	Change from baseline, mean (95% CI)	n	Mean (95% CI)	Change from baseline, mean (95% CI)		
Primary endpoint									
NRS pain	0	21	4.10 (2.98 to 5.21)		20	3.40 (2.25 to 4.55)			
	4	14	2.99 (1.69 to 4.28)	−1.11 (−2.42 to 0.20)	15	2.75 (1.49 to 4.01)	−0.65 (−1.93 to 0.63)	−0.46 (−−2.29 to 1.37)	.619
	8	16	2.15 (0.91 to 3.38)	−1.95 (−3.20 to −0.70)^b^	20	2.75 (1.60 to 3.90)	−0.65 (−1.81 to 0.51)	−1.30 (−3.01 to 0.41)	.135
	12	17	2.07 (0.86 to 3.28)	−2.03 (−3.25 to −0.80)^c^	19	3.32 (2.15 to 4.48)	−0.08 (−1.27 to 1.10)	−1.94 (−3.65 to −0.24)^a^	.026
NRS numbness	0	21	5.14 (3.96 to 6.32)		20	5.05 (3.84 to 6.26)			
	4	14	4.92 (3.62 to 6.21)	−0.23 (−1.27 to 0.82)	15	3.65 (2.37 to 4.93)	−1.40 (−2.41 to −0.38)^b^	1.17 (−0.29 to 2.63)	.114
	8	16	3.78 (2.53 to 5.04)	−1.36 (−2.36 to −0.36)^b^	20	3.30 (2.09 to 4.51)	−1.75 (−2.67 to −0.83)^c^	0.39 (−0.97 to 1.75)	.568
	12	17	3.71 (2.47 to 4.96)	−1.43 (−2.41 to −0.45)^b^	19	4.14 (2.92 to 5.36)	−0.91 (−1.84 to 0.03)+	−0.52 (−1.87 to 0.83)	.448
NRS tingling	0	21	4.33 (3.09 to 5.58)		20	3.50 (2.22 to 4.78)			
	4	14	3.59 (2.23 to 4.95)	−0.75 (−1.84 to 0.35)	15	3.05 (1.70 to 4.40)	−0.45 (−1.51 to 0.61)	−0.29 (−1.82 to 1.23)	.704
	8	16	3.18 (1.86 to 4.51)	−1.15 (−2.19 to −0.10)^a^	20	2.60 (1.32 to 3.88)	−0.90 (−1.86 to 0.06)+	−0.25 (−1.67 to 1.17)	.729
	12	17	3.28 (1.97 to 4.59)	−1.05 (−2.08 to −0.03)^a^	19	3.73 (2.44 to 5.01)	0.23 (−0.75 to 1.20)	−1.28 (−2.69 to 0.14)+	.076
Secondary endpoint									
FACT/GOG-Ntx subscale	0	21	26.10 (22.23 to 29.96)		20	29.05 (25.09 to 33.01)			
	4	13	29.39 (25.36 to 33.42)	3.29 (1.19 to 5.40)^b^	15	29.15 (25.10 to 33.20)	0.10 (−1.88 to 2.09)	3.19 (0.30 to 6.08)^a^	.031
	8	16	30.34 (26.38 to 34.30)	4.25 (2.29 to 6.20)^c^	19	30.41 (26.43 to 34.40)	1.36 (−0.47 to 3.19)	2.88 (0.20 to 5.56)^a^	.035
	12	17	30.37 (26.43 to 34.31)	4.28 (2.36 to 6.19)^c^	19	29.29 (25.31 to 33.27)	0.24 (−1.59 to 2.07)	4.03 (1.38 to 6.68)^b^	.003
Functional reach (cm)	0	21	33.22 (29.33 to 37.11)		20	33.9 (29.91 to 37.92)			
	4	12	37.92 (33.43 to 42.39)	4.69 (0.86 to 8.53)^a^	13	30.33 (25.91 to 34.72)	−3.63 (−7.34 to 0.08)+	8.33 (2.97 to 13.6)^b^	.003
	8	16	40.36 (36.12 to 44.55)	7.14 (3.68 to 10.59)^c^	17	32.31 (28.17 to 36.45)	−1.65 (−5.00 to 1.72)	8.79 (3.94 to 13.61)^c^	.001
	12	14	41.58 (37.26 to 45.89)	3.29 (8.35 to 11.98)^c^	17	34.54 (30.40 to 38.68)	0.58 (−2.77 to 3.96)	7.75 (2.79 to 12.7)^b^	.003
Chair to stand (seconds)	0	18	12.66 (10.87 to 14.45)		18	13.13 (11.37 to 14.88)			
	4	11	9.94 (8.01 to 11.87)	−2.72 (−4.03 to −1.42)^c^	12	12.13 (10.27 to 14.00)	−0.99 (−2.26 to 0.28)	−1.73 (−3.55 to 0.09)+	.062
	8	14	9.01 (7.14 to 10.87)	−3.65 (−4.86 to −2.45)^c^	17	11.58 (9.80 to 13.35)	−1.55 (−2.67 to −0.43)^b^	−2.11 (−3.75 to −0.46)^a^	.013
	12	10	8.03 (6.07 to 9.99)	−4.63 (−5.99 to −3.28)^c^	16	10.55 (8.76 to 12.34)	−2.58 (−3.72 to −1.43)^c^	−2.06 (−3.84 to −0.28)^a^	.024
No. of falls	0	18							
	4	11	0		12	0			
	8	14	2 (11.8%)		17	0			.23
	12	10	2 (14.3%)		16	2 (11.1%)			1
4-Meter walk speed (meters/second)	0	19	1.13 (1.01 to 1.26)		19	1.02 (0.90 to 1.15)			
	4	12	1.10 (0.95 to 1.24)	−0.04 (−0.17 to 0.09)	13	1.05 (0.91 to 1.19)	0.03 (−0.10 to 0.15)	−0.06 (−0.24 to 0.11)	.471
	8	15	1.16 (1.03 to 1.29)	0.03 (−0.09 to 0.14)	17	1.11 (0.98 to 1.23)	0.08 (−0.03 to 0.19)	−0.06 (−0.22 to 0.11)	.499
	12	14	1.18 (1.04 to 1.31)	0.04 (−0.08 to 0.16)	16	1.19 (1.06 to 1.32)	0.16 (0.05 to 0.28)^b^	−0.12 (−0.28 to 0.05)	.164

a CI = confidence interval; FACT/GOG-Ntx = Functional Assessment of Cancer Therapy/Gynecologic Oncology Group-Neurotoxicity; NRS = numeric rating scale; UC = usual care.

^b^ *P* < .05. ^c^*P* < .01. ^d^*P* < .001.

### Functional Outcomes

**Figure 4.**
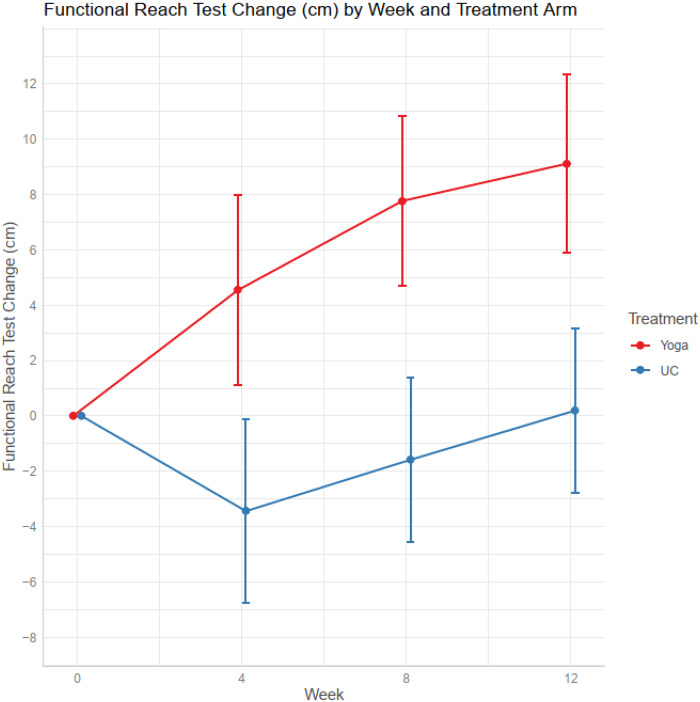
Changes in Functional Reach Test by weeks. UC = usual care.

Yoga was more effective than usual care at improving functional reach (Figure 4 and Table 2). The mean functional reach was similar in the 2 treatment groups at baseline; by 8 weeks it has increased by 7.14 cm (95% CI = 3.68 to 10.59) in the yoga group and decreased by 1.65 cm (95% CI = -5.00 to 1.72) in the usual care group (*P *=* *.001 for the difference between groups). The chair to stand time decreased 3.65 seconds (95% CI = -4.86 to -2.4) in the yoga group compared with 1.55 seconds (95% CI = -2.67 to -0.43) in the usual care group (*P* = .013 for the difference between groups). There were no statistically significant differences in the 4-meter walk speed test or numbers of falls between the 2 arms (Table 2).

### Pain Medication Usage

Yoga and usual care had similar pain medication usage at baseline (28.5% and 30.0%, respectively). At 8 weeks, relatively fewer yoga patients reported using pain medication (19%) compared with usual care patients (35%), but this difference was not statistically significant (*P* = .44). Similarly, at 12 weeks, 25% of yoga patients and 35% of usual care patients reported pain medication usage (*P* = .71).

### Adverse Events

Three out of 21 patients experienced 4 grade 1 yoga-related adverse events. These included 3 incidents of myalgia and 1 leg cramp. No adverse events were reported in the usual care group.

## Discussion

CIPN is a debilitating and persistent condition that substantially worsens cancer survivors’ quality of life and increases the risk of falls. We previously found that 58.4% of breast cancer survivors who received taxane-based chemotherapy experienced persistent neuropathic symptoms for a mean duration of 5.6 years after completing chemotherapy (58). In this randomized clinical trial of breast and gynecologic cancer patients with moderate to severe CIPN, yoga was shown to be not only safe but also potentially effective in improving CIPN-related pain and functional outcomes.

To the best of our knowledge, our study is the largest randomized usual care controlled trial to show the promising efficacy of yoga in improving CIPN-related pain and functional outcomes in breast and gynecological cancer survivors. Only 2 prior pilot trials have explored the effects of yoga on CIPN. Our results are consistent with a previous 10-patient, single-arm study that found yoga reduced CIPN pain by 1.94 points (*P* = .04) on the Brief Pain Inventory (0-10 scale) (25). Our findings are also in line with a 26-patient, 4-arm randomized controlled trial (7 patients in the yoga arm) that found yoga improved the FACT/GOG-Ntx score by 2.12 points though without statistical significance compared with an educational control potentially because of the small sample size of the study (24).

The magnitude of effect of yoga on CIPN pain found in our study is clinically meaningful. Currently the only intervention for CIPN supported by the American Society of Clinical Oncology guidelines is duloxetine, which was shown to achieve a statistically and clinically meaningful reduction in CIPN-related pain by 1.06 points on the Brief Pain Inventory. However, duloxetine use was associated with side effects such as nausea, headache, and fatigue, demonstrated by the high 12% dropout rate in the duloxetine arm compared with a 1% dropout rate in the placebo arm (59). Though preliminary, yoga was found to reduce CIPN pain by 1.95 points after 8 weeks and 2.03 points after 12 weeks. The proportion of patients in the yoga arm on pain medication reduced from 30.0% at baseline to 18.8% at week 8. Pain reduction exhibited in the yoga arm was nonetheless of greater magnitude when compared with usual care with minimal side effects. This suggests that yoga is a promising nonpharmacological approach to improve CIPN pain and highlights the importance of further research with a larger sample size and a longer follow-up period.

Our study demonstrated that yoga improves not only CIPN pain but functional outcomes as well. The 4.24-point improvement in the FACT/GOG-Ntx score is in line with the minimal clinically important difference reference of a 3.3-4.4 point change based on recommendations from the FACT/GOG-Ntx subscales (60). Compared with usual care, 8 weeks of yoga also statistically significantly improved functional reach by 7.14 cm and shortened chair to stand time by 3.65 seconds. The Functional Reach Test has demonstrated clinical validity to be a strong predictor of fall recurrence with functional reach less than 25.40 cm to predict double the fall risk, functional reach less than 15.24 cm to predict quadruple the fall risk, and no reach ability to indicate an eightfold likelihood of falling (47). The participants in our study carried a relatively high functional status going into the study with an average functional reach greater than 25.4 cm at baseline. Nonetheless, the yoga group still achieved a greater improvement in functional reach compared with usual care. Additionally, chair to stand results demonstrated a similar trend, with yoga statistically significantly reducing chair to stand time compared with usual care. As functional reach and chair to stand are predictive of the risk of falls, our results show promising signals of yoga in reducing CIPN-related fall risks and warrant further definitive studies. Our study also demonstrated a high study adherence rate. Although the dropout rate in the yoga group is slightly greater than usual care, the reasons for dropping out were mainly personal rather than because of intolerance of the yoga regimen.

While the exact mechanism of yoga on CIPN improvements is unknown, 2 hypotheses have been proposed. The first postulates that yoga increases blood flow and oxygen supply to provide neuroprotective effects (61). The second suggests that the mindfulness and breathing exercises in yoga upregulate the parasympathetic nervous system while mediating sympathetic activities through the hypothalamic-pituitary-adrenal axis (62). It has yet to be discerned which aspect of yoga—*asanas*, *pranayama*, or meditation—has the greatest effect on CIPN, or if the impact is due to a combination of these elements. A previous large study found that 6 weeks of moderate intensity exercise statistically significantly improved CIPN symptoms of numbness and tingling (63). Heterogeneity of yoga interventions (eg, selected *asanas*, length of practice or intervention, adherence) may partially explain observed differences in outcomes across studies. Standardization of yoga protocols may improve our understanding of the effect of yoga and its possible mechanisms, as well as the applicability of this research.

Our study is limited by a modest sample size, a lack of objective measurements in CIPN symptoms, and lack of evaluator blinding. Additional limitations include the use of self-reported outcomes, which is subjected to reporting bias. Our participants also received different types of chemotherapy; however, most patients received paclitaxel therapy. We also measured multiple outcomes; these were meant to be exploratory and should be confirmed in future trials to adequately verify the novel findings. However, this study was strengthened by well-balanced patient characteristics between groups at baseline, as well as a high adherence rate, with 88% of patients completing all study-related requirements. Though our yoga protocol is 4-6 weeks longer than previously reported studies, our adherence rate is higher than the 40% to 61% reported rates in previous studies (24,25).

Among breast and gynecological cancer survivors with moderate-to-severe CIPN, yoga appeared to be safe and showed promising effect in improving CIPN-related pain, quality of life, and physical functioning outcomes. An adequately powered randomized controlled trial with a larger sample size, longer follow-up, and appropriate control is warranted to confirm the specific and long-term effects of yoga on CIPN symptoms and fall prevention.

## Funding

This work was supported in part by a National Institutes of Health/National Cancer Institute Cancer Center grant (grant number P30 CA008748); the Translational and Integrative Medicine Research Fund at Memorial Sloan Kettering Cancer Center; and the Frueauff Foundation.

## Notes

**Role of the funder:** The funding sources were not involved in the study design; collection, analysis, and interpretation of data; writing of the report; or decision to submit the article for publication.

**Acknowledgments:** The authors would like to extend their gratitude to yoga instructors Clare Patterson and Tina Paul for providing the interventions for this study; Patricia Chen for her assistance in conducting the study, and Christina Seluzicki for editorial support. The authors would also like to thank all the cancer survivors in this study for their participation. Dr Ting Bao (the principal investigator) had full access to all the data in the study and takes responsibility for the integrity of the data and the accuracy of the data analysis.

**Disclosures:** Dr Bao was a medical advisor for Eisai. Dr Mao reports grants from Tibet Cheezheng Tibetan Medicine Co., Ltd and Zhongke Health International LLC outside the submitted work. We certify that there are no affiliations with or involvement in any organization or entity with any financial interest or other equity interests or non-financial interests that influenced the design, outcome, and submission of this study.

**Author contributions:** TB and JM: Conception and design. TB, IZ, VB, MR, AS, and JM: Provision of study material or patients. LP and QSL: Collection and assembly of data. TB, IZ, RB, MH, CC, MLG, KP, and JM: Data analysis and interpretation. All authors: Manuscript writing. All authors: Final approval of manuscript. All authors: Accountable for all aspects of the work.

## Data sharing statement

After de-identification, individual participant data that underlie the results reported in this article, the study protocol, and the statistical analysis plan will be available beginning 3 months and ending 36 months following article publication to investigators whose proposed use of the data has been approved by an independent review committee identified for this purpose for individual participant data meta-analysis. Proposals should be directed to baot@mskcc.org to gain access, data requestors will need to sign a data access agreement.
